# Future Perspectives of Oral Delivery of Next Generation Therapies for Treatment of Skin Diseases

**DOI:** 10.3390/pharmaceutics13101722

**Published:** 2021-10-18

**Authors:** Pia Pernille Søgaard, Marianne Lind, Chatpakorn Rassemeena Christiansen, Karsten Petersson, Adam Clauss, Ester Caffarel-Salvador

**Affiliations:** 1 Regenerative Medicine Department, LEO Pharma A/S, Industriparken 55, 2750 Ballerup, Denmark; ZGDDK@leo-pharma.com (P.P.S.); CHRCI@leo-pharma.com (C.R.C.); AZCDK@leo-pharma.com (A.C.); 2 Explorative Formulation and Technologies, LEO Pharma A/S, Industriparken 55, 2750 Ballerup, Denmark; MMDDK@leo-pharma.com (M.L.); KAPDK@leo-pharma.com (K.P.); 3 LEO Science & Tech Hub, One Broadway, Cambridge, MA 02142, USA

**Keywords:** skin diseases, oral biologics, gene therapy, oligonucleotides, CRISPR, oral devices, drug-device combinations, clinical trials, epidermolysis bullosa, regenerative medicine

## Abstract

Gene therapies have conspicuously bloomed in recent years as evidenced by the increasing number of cell-, gene-, and oligo-based approved therapies. These therapies hold great promise for dermatological disorders with high unmet need, for example, epidermolysis bullosa or pachyonychia congenita. Furthermore, the recent clinical success of clustered regularly interspaced short palindromic repeats (CRISPR) for genome editing in humans will undoubtedly contribute to defining a new wave of therapies. Like biologics, naked nucleic acids are denatured inside the gastrointestinal tract and need to be administered via injections. For a treatment to be effective, a sufficient amount of a given regimen needs to reach systemic circulation. Multiple companies are racing to develop novel oral drug delivery approaches to circumvent the proteolytic and acidic milieu of the gastrointestinal tract. In this review, we provide an overview of the evolution of the gene therapy landscape, with a deep focus on gene and oligonucleotide therapies in clinical trials aimed at treating skin diseases. We then examine the progress made in drug delivery, with particular attention on the peptide field and drug-device combinations that deliver macromolecules into the gastrointestinal tract. Such novel devices could potentially be applied to administer other therapeutics including genes and CRISPR-based systems.

## 1. An Introduction to the Next Generation of Treatments

### 1.1. Oligonucleotides

Oligonucleotides are nucleic acid chains formed by the polymerization of nucleotides linked by a phosphodiester backbone. These molecules provide an excellent opportunity for use in therapeutic interventions due to their rational design and accessibility to targets unavailable to conventional pharmaceuticals [1,2,3]. Although various applications for therapeutic oligonucleotides exist, the common mechanism of action is based on either the complementary Watson–Crick base pairing between the drug and the target mRNA or protein interaction through a three-dimensional structure [2,3,4,5].

Gene regulation is an application for antisense oligonucleotides (ASO) and RNA interference (RNAi) in which the oligonucleotide binds to its designated target that contains a complementary sequence [2,3,4]. For ASOs, binding of the target mRNA by a single-stranded oligonucleotide can either facilitate ribonuclease-mediated degradation or cause a steric blockage that disrupts RNA–RNA and/or RNA–DNA interaction, thereby interfering with RNA splicing [4,5]. In contrast, RNAi relies on the introduction of RNA molecules including small interfering RNA (siRNA) and microRNA (miRNA) into the cell cytoplasm where the guide strand is incorporated into the RNA-induced silencing complex (RISC), which can then target mRNA for degradation [3,6]. Another approach that also functions through complementary binding is a class of catalytically active oligonucleotides called DNAzymes. These single-stranded molecules catalyze a variety of biochemical reactions such as site-specific RNA cleavage, DNA cleavage, RNA/DNA ligation and DNA phosphorylation [7,8,9]. A different approach for therapeutic nucleotides includes a class that functions through protein–nucleic acid ligand interaction. This class is called aptamers and consists of a single-stranded oligonucleotide that conforms into a defined three-dimensional structure that can bind a protein target through adaptive fit [2,10,11].

**Figure 1:**
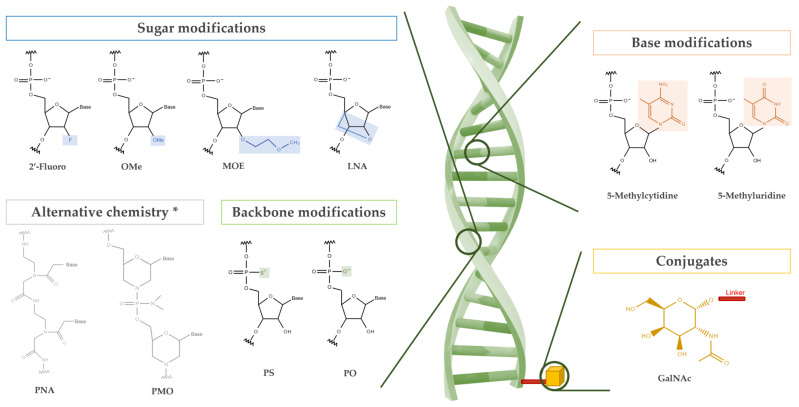
Schematic depicting the most commonly used oligonucleotide modifications. OMe: 2′-O-methyl, MOE: 2′-methoxyethyl, LNA: locked nucleic acid, PS: phosphorothioate, PO: phosphorodiester, PNA: peptide nucleic acid, PMO: phosphorodiamidate morpholino oligomer, GalNAc: N-acetylgalactosamine. * These chemistries are used in single stranded ASOs.

Despite the existing variety of oligonucleotides and their rising popularity, natural oligonucleotides suffer from inadequate physicochemical properties that results in low efficiency *in vivo* [12,13]. To circumvent these flaws, they are enhanced with chemical modifications. These modifications can be broadly categorized into backbone, base, sugar, and conjugate modifications on the oligonucleotide (Figure 1) [3,14,15,16]. Commonly known and used modifications include phosphorothioate, 2′-O-methyl, and locked nucleic acids to address stability, toxicity, and nuclease resistance while conjugations like N-acetylgalactosamine (GalNAc) have been used to direct targeted tissue delivery [2,3,4,14,17,18].

### 1.2. DNA Editors

Gene editing has gained extensive attention as a strategy to correct disease-causing mutations in the genome. With the advent of clustered regularly interspaced short palindromic repeats (CRISPR) technology for editing in mammalian cells, CRISPR-based methods have widely replaced the earlier and more labor-intensive methods for gene editing such as transcription activator-like effector nucleases (TALENs) and zinc-finger nucleases (ZFNs) [19]. CRISPR-based gene editing has since proven a versatile technology with great impact as a tool in biological research in addition to potential therapeutic applications ranging from editing of small disease-causing mutations to excision and replacement of disease-causing genes with functional copies. CRISPR-based gene editing is a rapidly evolving field of research that has given rise to several new technologies including CRISPR associated protein 9 (CRISPR-Cas9), CRISPR base editors, and prime editors [19,20,21].

CRISPR-Cas9 mediated gene editing builds on the principle of having a nuclease capable of creating double strand breaks (DSBs) being targeted to a specific sequence in the genome by forming a complex with a guide RNA (gRNA) [22]. Once the gRNA-Cas9 has bound its target site, Cas9 will introduce DSB, triggering endogenous DNA repair pathways which, in turn, cause the editing events. As such, the non-homologues end-joining (NHEJ) repair pathway can lead to disruption of gene function by the introduction of insertions or deletions in the gene sequence, whereas the homology-directed repair (HDR) pathway can be exploited for the introduction of a new sequence by providing the cells with a donor DNA template encoding the desired sequence to be inserted [22]. Such HDR-mediated insertion is inefficient compared to NHEJ, and the introduction of a DSB can lead to unwanted indel mutations, making this technology less suitable for correcting single base mutations in a therapeutic context [21]. A strategy to circumvent this problem has arisen with base editors, which allow for single base corrections without DSB induction [21,23,24]. This approach can be used to correct or introduce point mutations which, in addition, can be used to create premature stop-codons, thereby silencing the gene [24,25]. Base editing has proven to be a more efficient process for base modification compared to CRISPR-Cas9 with HDR and relies on the principle of fusing a catalytically dead Cas9 (dCas9) to a DNA modifying enzyme such as a deaminase [21]. The activity of the DNA-modifying enzyme is then directed by the gRNA to a specific location in the genome where it introduces a point mutation. As an example, a deaminase like tadA can convert an adenine (A) to a guanine (G) by deaminating adenosine into inosine. Because inosine is read as a guanosine during DNA replication, this leads to an A to G point mutation. Newer generations of base editors have been developed to increase the repertoire of possible base changes as well as enhancing the editing efficiency [21,23]. More recently, prime editing has emerged as a method to directly write new genetic code into the genome in a “search and replace” manner. With this methodology, a specialized gRNA contains both a sequence for targeting the complex to the right sequence and a template for the desired edit. By including an engineered reverse transcriptase in the complex, this RNA template allows for programmable edits to be incorporated into the genome [26].

Base editing holds great potential for therapeutic applications, but it has not yet been tested in a clinical setting. In contrast, traditional CRISPR editing is increasingly being taken forward to clinical applications. The delivery of CRISPR-based technology remains a major challenge for clinical translation. The need for biocompatible vehicles to encapsulate such technology and to achieve systemic delivery with tissue specificity are the main obstacles [27]. Furthermore, the immunogenicity of the bacterially derived Cas9 protein adds to this challenge [28]. Thus, it is not surprising that the main focus so far has been on *ex vivo* editing of patient cells. Nevertheless, a recent phase I study has shown a promising safety profile for *in vivo* editing of the transthyretin (TTR) gene for the treatment of transthyretin amyloidosis (ATTR) [29]. ATTR is a fatal disease caused by misfolded TTR protein forming amyloid fibrils which accumulate in tissues and cause polyneuropathy and cardiomyopathy. In this study, six patients were dosed intravenously with NTLA-2001, a CRISPR-Cas9 based gene-editing therapy, formulated in nanoparticles with tropism for the liver, where >99% of TTR protein is produced [29]. Results from this study showed an encouraging safety profile with only mild adverse events reported. Successful gene editing was reflected by TTR concentrations in the serum decreasing by 47–56% and 80–96% in the lower and higher dose group, respectively [29]. Proof-of-concept for symptom relief and functional improvement upon the reduction in TTR protein in the serum of ATTR patients has previously been provided by Patisiran, an siRNA-based therapy that transiently reduces TTR expression by degradation of TTR-encoding mRNA [30]. Therefore, the above results indicate that a permanent reduction of TTR could improve patient outcome without the requirement for life long repetitive administration and associated side effects.

Gene editing and delivery as well as oligonucleotide-based therapies have conspicuously evolved addressing unmet patient needs in different fields. While oncology and hematology lead the list of fields where these therapies are being applied, dermatology also takes a stake centering on rare diseases [31]. The next section compiles such dermatological applications that have reached the clinic.

## 2. Gene Therapy in Dermatology

### 2.1. Gene Therapy and Gene Editing

Gene therapies have not yet been approved to treat skin diseases but the progress in the field has been remarkable over the last couple of decades. For context, China was the first country to approve and commercialize a gene therapy product back in 2003 [32]. It was not until 2012 that Glybera became the first gene therapy approved in Europe by the European Medicines Agency (EMA) for the treatment of lipoprotein lipase deficiency, an ultra-rare inherited disorder [33]. Five years later, the United States Food and Drug Administration (FDA) gave approval to Luxturna, ushering in a new range of possibilities for disease treatment [34]. Today, there are close to a dozen gene therapy products approved by the different regulatory agencies, and this number is on the rise [4,35].

Progress on gene editing and gene delivery in dermatology is evidenced by a number of pre-clinical and clinical reports [36]. The biggest strides made in clinical studies by gene therapy in dermatology have been toward epidermolysis bullosa (EB), followed by melanoma and other rare diseases like Netherton syndrome and congenital ichthyosis [37,38,39]. EB is a family of rare genetically heterogenous disorders that cause fragility and blistering of the skin and mucous membranes. Its severity ranges from mild to fatal. Junctional EB (JEB) is the most severe subtype caused by the absence of anchoring proteins due to mutations in the laminin 322 genes (*LAMA3*, *LAMB3*, and *LAMC2*) [40]. The skin blistering characteristic of dystrophic EB (DEB) is ascribable to mutations in the gene that encodes for type VII collagen, *COL7A1* [41]. Within the DEB spectrum, recessive DEB (RDEB) is the most severe form with blisters spread over the whole body also affecting mucous membranes in the gastrointestinal tract. Milder symptoms of this condition are noted for dominant DEB (DDEB), primarily localized on the hands, elbows, feet, and knees. Efforts have been made to deliver the 8833-nucleotide open reading frame of the *COL7A1* gene, to DEB patients to restore the production of type VII collagen [42]. Nonetheless, the large size of the gene hinders its packaging into viral vectors and limits the transduction efficiency in addition to reducing the viral titer. Most of the gene therapy research has been *ex vivo*, focused on cell culture and transplantation of cultured skin grafts [43,44]. Many groups initially explored the delivery of *COL7A1 ex vivo* using a retrovirus to correct primary patient keratinocytes that were later xenografted onto immunodeficient mice [45,46]. Since then, the number of gene therapy approaches that have made it to the clinic has increased. Recorded clinical trials using gene therapy for EB and other skin conditions are compiled in Table 1.

Genetically engineered autologous skin equivalent grafts dominate the EB treatment landscape in clinical trials, as shown in Table 1. A notable example of gene editing in dermatology is the restoration of the skin by autologous, transgenic epidermal grafts on approximately 80% of the total body surface area of a 7-year-old patient suffering from JEB [49]. In this report, keratinocytes derived from the patient were transduced with a functional *LAMB3* gene *ex vivo* using a retroviral vector to generate permanently edited keratinocytes. Epidermal grafts were created from the transgenic cells and used to transplant onto the patient, thereby restoring the skin of the patient, which resulted in a sustained and robust epidermis throughout the 21-month follow-up period [49]. The potential for long term benefits from grafted transgenic epidermis was demonstrated in another study using a similar approach on a smaller area of the body of a JEB patient. Here, the positive effect of treatment was still present 6.5 years later [50]. Castle Creek Biosciences’ D-Fi (debcoemagene autoficel), named FCX-007 prior to Fibrocell Science acquisition, is comprised of autologously-derived fibroblasts from RDEB patients genetically corrected using a lentiviral vector encapsulating the *COL7A1* gene. These genetically modified fibroblasts are then intradermally injected to the patient at the wound site. Wound healing has been observed to last for up to a year using the D-Fi treatment [51]. D-Fi has received orphan drug designation by the FDA for DEB. Unlike D-Fi, EB-101 from Abeona Therapeutics consists of autologous RDEB keratinocytes—instead of fibroblasts—isolated from skin biopsies but transduced with a recombinant retrovirus—instead of a lentiviral vector—containing *COL7A1* [52,53]. Abeona Therapeutics just released promising results from their phase I/IIa clinical study following treatment with EB-101 for RDEB. Wound healing of at least 50% was observed in approximately 70% of the wounds after three years and 80% at year six. Absence of pain was recorded for all treated wounds. EB-101 phase III results on large chronic wounds are due in mid-2022 [52]. Other studies, however, have suggested that, for successful anchoring fibril formation, type VII collagen needs to be expressed in both fibroblasts and keratinocytes [54].

Netherton syndrome, a rare skin disease caused by loss of function mutations in the *SPINK5* gene, has also been the target of autologous skin grafts pre-clinically and on a phase I clinical trial [55,56,57]. The *SPINK5* gene encodes for the lymphoepithelial Kazal-type-related inhibitor (LEKTI) responsible for the regulation of skin desquamation. In a clinical trial, a de-epidermized skin lesion was treated with lentiviral gene therapy to restore the function of LEKTI. Unfortunately, despite having used an integrative vector, LEKTI expression was transient and did not sustain past three months [57]. Krystal Biotech is investigating the potential of topically administering the *SPINK5* gene via non-integrating herpes simplex virus type 1 (HSV1) vectors to treat Netherton’s syndrome [58].

Besides questions to the feasibility and practicality of grafting *ex vivo* cultured grafts onto substantial areas of the body of patients, the permanent delivery of a gene into the genome presents concerns over insertional mutagenesis stemming from integration of the gene into an undesirable location in the genome. The latter concerns may be addressed by editing the mutated gene using CRISPR-based strategies, these, however, fail to address the large practical challenge and cost of grafting. Furthermore, CRISPR-mediated editing would in many cases need to be tailored to individual patient mutations which, particularly in the context of rare diseases, makes it challenging to translate into viable clinical therapies.

As an alternative approach to permanent gene delivery, the potential to transiently deliver functional genes to the skin *in vivo* is also currently being pursued. Examples of this include Krystal Biotech’s phase I/II study where functional *COL7A1* was delivered to the wound bed of RDEB patients using an attenuated, non-replicating HSV1 viral vector (B-Vec) [59]. Data from these initial studies showed wound closure in 90% of treated wounds. Subsequently, recruitment for an ongoing phase III study (NCT04491604) was completed in March 2021 [59]. A major concern with viral delivery is the possibility of immunological responses, particularly in the light of transient therapy that would need repeated dosing throughout the patient lifespan. An attractive, yet less explored, path thus remains transient, non-integrating gene delivery using non-viral delivery vectors. Preclinical work from Amryt Pharma shows that delivery of the *COL7A1* gene formulated in highly branched poly β-amino ester–AP103 can achieve five times higher expression of type VII collagen in RDEB keratinocytes compared to healthy keratinocytes [60]. When applied topically to human RDEB skin grafted onto mice, type VII collagen was observed at the interface between the dermis and the epidermis up to 10 weeks after treatment. A phase I/II for AP103 is scheduled for 2022 [61].

In multiple *ex vivo* studies, CRISPR/Cas9 has been harnessed to correct the *COL7A1* mutation in induced pluripotent stem cells derived from patients with RDEB with subsequent transplantation onto mice of the skin equivalents grown from corrected keratinocytes [38,39,62,63,64]. CRISPR-based gene editing is also being explored for non-rare dermatological conditions with a recent study demonstrating *in vivo* delivery of ribonucleoprotein using microneedles in mouse models of atopic dermatitis and psoriasis [65]. For a more comprehensive review of gene editing and gene therapy in the context of dermatology, we refer the reader to these excellent recent reviews [66,67]. Furthermore, mesenchymal stromal/stem cells derived from skin or bone marrow are being evaluated in clinical trials in adults and children suffering from RDEB [68,69,70]. Intravenously administered recombinant type VII collagen protein replacement is also under investigation in a phase II clinical trial (NCT04599881) on RDEB patients [71]. The advances on the dermatological field using stem cell therapies are notable, enabling tissue regeneration or tissue damage correction at the genetic level [49,72,73]. While protein replacement and cell therapy approaches are beyond the scope of this review, multiple research articles describe the treatment options for skin diseases including chronic auto-inflammatory diseases, EB, and would healing [73,74,75,76].

### 2.2. Oligonucleotide Therapies in Dermatology

The progress on oligonucleotide chemistry in the 1960s set a landmark on the evolution of oligonucleotides, which were already tested in clinical trials in the 1990s. Fomivirsen, delivered via injection to the eye, was the first ASO approved by the FDA in 1998 for the treatment of cytomegalovirus-induced retinitis [77]. Twenty years later, in 2018, the same agency gave global approval to the first siRNA therapy, Patisiran, for the treatment of TTR, as described earlier. It was encapsulated into lipid nanoparticles for hepatocyte delivery [78,79]. Today, over a dozen of oligonucleotide therapeutics have already been approved by the FDA to treat several indications caused by single gene mutations [80]. This has prompted a surge of research focused on oligonucleotides for the treatment of rare diseases. There are over 85 and 115 registered clinical trials in the U.S. with a focus on siRNA and ASOs, respectively. A compilation of the ongoing and completed clinical trials in the dermatological field, excluding skin melanoma and skin wounds, is exhibited in Table 2.

Pachyonychia congenita, an ultra-rare autosomal dominant disorder resulting from a mutation in one of the keratin genes (*KRT6A*, *KRT6B*, *KRT6C*, *KRT16*, or *KRT17*), was the first inherited skin disorder to be targeted using oligonucleotide therapeutics in humans. Specifically, the patient was treated with an siRNA targeting the N171K mutation characterized by the cytosine-to-adenine single nucleotide K6a mutation [81]. This selective mutation depletion seemed to be the trigger of the callus reduction observed in the trial, hence, the use of siRNA has the potential of correcting the molecular etiology of the disease.

Hypertrophic scarring is another dermatological condition highly targeted with oligonucleotides. It consists of pathological thickened and elevated scars resulting in a collagen imbalance at the wound site. No pharmaceuticals have yet been approved by the FDA or EMA to treat such a disease, but several RNAi regimens are being investigated at the bench and in the clinic [82,83]. Amongst these, several siRNA treatments stand out in clinical trials, as shown in Table 2. The first one to note is the asymmetric siRNA from Olix Pharmaceuticals with proven efficient gene regulation. OLX10010, Olix Pharmaceuticals siRNA candidate, is being tested in a phase II trial for the treatment of hypertrophic scarring by intradermal injection and Hugel has acquired its exclusive sales rights to treat this condition in Asian countries [84]. STP705, Sirnaomics’ lead candidate, is another siRNA that diminishes both inflammation and fibrotic activity. It is being investigated for both hypertrophic scar reduction and skin squamous cell carcinoma by intradermal or intralesional injection. This siRNA is delivered encapsulated into Sirnaomics’ proprietary polypeptide nanoparticles consisting of a branched histidine lysine polypeptide, which confer protection to the siRNA and enable delivery to the targeted body cells [85]. The potential of the self-delivering RNAi platform developed by RXi Pharmaceuticals, now Phio Pharma, is being evaluated for its ability to reduce dermal scarring, also known as fibrosis, by silencing the connective tissue growth factor. This hybrid oligonucleotide leverages the advantages of both antisense technologies and RNAi enabling target specificity, efficient cellular uptake, high potency, and serum stability while minimizing the immune response activation [86,87]. Pfizer’s ASO PF-06473871, however, was abandoned during clinical development because despite showing a successful inhibition of the scarring process, reducing scar severity, it did not significantly outperform the surgical approach [83].

Several preclinical studies have validated the essential role that GATA3 (GATA binding protein 3), a transcription factor, plays in inflammatory disorders. Specifically, decreased expression of GATA3 enhances skin inflammation in many chronic inflammatory diseases including psoriasis and atopic dermatitis [88,89]. GATA3 is responsible for the production of key inflammatory cytokines including interleukin-13 (IL-13), which mediate inflammation. The overproduction of IL-13 is one of the mainsprings of the pathogenesis of atopic dermatitis. Consequently, inhibiting the function of either IL-13 or GATA3 can be a strategy to treat inflammatory diseases. This aim was pursued by Sterna biologics, a pharmaceutical company that made GATA3 a druggable target using the active pharmaceutical ingredient hgd40. Hgd40 is a catalytic ASO, namely a DNAzyme, which specifically cleaves GATA3 mRNA averting immune system activation. Sterna’s DNAzyme asset completed phase IIa clinical trials with a topical formulation as a proof of concept for atopic dermatitis and is being pursued for other indications outside dermatology [90].

ASOs have been widely explored to restore gene functionality and, in turn, evoke disease correction [91,92,93]. Exon skipping induced by ASOs provides a way to modulate the pre-mRNA splicing process to eliminate a mutated exon from the mature mRNA. Exons 73, 80, and 105 in the *COL7A1* gene are known to harbor recurrent mutations in RDEB. The dispensability of these pathogenic mutation-containing exons can restore the function of type VII collagen, shifting the severity of the disease phenotype. Pre-clinical studies in primary RDEB fibroblasts and keratinocytes with mutations in exons 73 and/or 80, transfected with 2’-O-methyl antisense oligoribonucleotides, have demonstrated the potential of exon skipping to restore type VII collagen expression and the formation of anchoring fibrils [93]. In the clinic, phase I/II results are pending for the QR-313 asset by Wings Therapeutics, an exon skipping ASO comprised of 21 oligonucleotides. It has been topically administered to subjects with DDEB or RDEB with one or multiple mutations in the *COL7A1* gene [94]. QR-313 hybridizes to a specific sequence in the *COL7A1* pre-mRNA to exclude exon 73 from the mRNA. In a murine model of atopic dermatitis, where IL-13 is overproduced, an IL-13 ASO administered topically using liposomes significantly suppressed the IL-13 production up to 70% compared to the control group. Furthermore, it also reduced the infiltration of inflammatory cells, reducing the skin thickness [95]. Exicure’s (formerly AuraSense Therapeutics) ASO-based assets AST-005 and XCUR17 are products of a nanoparticle-based nucleic acid delivery platform called spherical nucleic acids (SNA™). These are formulated as a topical gel and have been evaluated in phase I clinical trials for psoriasis [96,97]. AST-005, tested in collaboration with Purdue Pharma, knocks down a tumor necrosis factor (TNF), a pro-inflammatory cytokine demonstrated to be a key psoriasis mediator. On the other hand, XCUR17 targets the IL-17 receptor alpha, an essential protein in the initiation and maintenance of psoriasis. Despite positive safety and tolerability results of SNA and reductions in the major psoriatic inflammatory markers, efficacy did not meet the expected statistically significance. Nonetheless, Allergan has partnered with Exicure to explore the potential of XCUR17 for alopecia, and Purdue retains the rights to further develop anti-TNF drug candidates. XCUR17 could also be used for the treatment of Netherton syndrome, an application that is still available for out-licensing [96,98]. Not to forget, ASOs have also been investigated in the clinic to treat multiple melanomas including skin melanoma, but as previously stated, cancers fall outside the scope of this manuscript [99].

### 2.3. Gene Delivery Challenges

The main challenge associated with gene therapies is delivery. The obstacles associated with the delivery of nucleic acids into targeted tissues are attributed to their low serum stability, size, charge, and immune system stimulation [100]. Moreover, oligonucleotides must be able to escape from the endosomes and enter the cytosol after being taken up by the cell. Delivery of plasmids, furthermore, needs to overcome the challenge of accessing the cell nucleus. To circumvent these challenges and guarantee a therapeutic effect, it is imperative to select an appropriate carrier. Viral and non-viral vectors have been investigated for both oligo and gene transfer as described in several recent reviews [101,102,103]. Herpes simplex virus, lentivirus, adenovirus, and adeno-associated virus (AAVs) are some of the most common viral vector candidates in addition to retroviruses, which so far have been the most frequently employed viral vectors for cutaneous gene transfer in the clinic (Table 1). Viral vectors, specifically AAVs, have extensively been studied since their discovery in 1965 for their ability to deliver a cargo to the nucleus of the cell. In 1995, AAVs were utilized in the first human application, and currently, several treatments leverage their delivery potential in the market [103]. AAVs have also been harnessed in pre-clinical studies for dermatological applications. An example of this is a study aiming to correct a mutation in the *COL7A1* gene in keratinocytes from a RDEB patient. In this study, AAVs and adenoviral vectors expressing donor template DNA and TALENs, respectively, were combined to correct gene function by restoring the reading frame through introduction of small insertions and deletions [104]. The limited packaging capacity of approximately 5 kb, the immune response evoked by viral backbones, and the high manufacturing costs, however, remain an obstacle for further expansion of gene therapy applications of viral vectors [105,106]. These are some of the reasons why non-viral delivery systems are gaining popularity for encapsulation and delivery. Lipid nanoparticles, polyplexes, nanospheres, dendrimers, and exosomes are some examples of non-viral vectors [2,107,108]. Nanoparticles are the most prevalent approach to circumvent the delivery problem. They are also being widely investigated for oral delivery with the goal to protect gene vectors from gastric acid degradation and to facilitate its transport across the intestinal epithelium [109]. Despite the fact that oral administration of gene therapies has not yet reached the clinic, multiple studies have validated its potential in preclinical studies [109,110,111,112,113].

Regardless of the method used for encapsulation and cell targeting, the route of administration has a great impact on therapy adherence rates. All the approved therapies based on oligonucleotides are administered via different types of injections, either intravitreal, subretinal, intrathecal, intravenous, intramuscular, or subcutaneous [4]. Considering the pain associated with the skin conditions described in this review, injections and the topical application of a therapy can exacerbate the discomfort. As a case in point, a patient receiving siRNA intradermal injections for the treatment of pachyonychia congenita reported intense injection-related pain. As a consequence, the patient had to be pre-treated with pain killers and anesthetics to mitigate the pain of the injections [81]. While the siRNA therapy led to disease regression, the pain of administration precluded its clinical translation, evidencing the need for alternative drug delivery regimens.

Topical delivery of nucleic acids has also been investigated to achieve local targeting of the skin [114,115,116]. Microneedles, a transdermal patch used to breach the *stratum corneum* in a minimally invasive manner, and electroporation alone and combined have successfully delivered gene therapies [117,118]. One example is the use of biocompatible microneedles to encapsulate and deliver CRISPR-Cas9 to treat inflammatory skin disorders [65]. However, microneedle delivery can be challenging with altered skin properties including open wounds characteristic of RDEB where the delivery may be inconsistent or extremely painful, respectively. On the other hand, the lack of the *stratum corneum* barrier in dermatological diseases characterized by wounds and skin lesions could be harnessed for topical delivery without the need for physical methods to disrupt the skin.

The route of administration has a direct impact on the clinical and commercial success of a given therapy. Many dermatological diseases are treated with macromolecules that need to be injected including peptides, proteins, and monoclonal antibodies. This includes 11 biologics approved by the FDA for the treatment of moderate to severe psoriasis. Injections pose a burden to many patients, revealing an underlying need to identify alternative drug administration options such as enabling absorption across the intestinal and skin barriers [119]. The oral route remains an attractive alternative for delivery, but despite being the preferred route of administration, it is often overlooked for gene delivery due to the susceptibility of nucleic acids to the intestinal environment. Seminal advances are key to overcoming such challenges, attaining new solutions for diseases with unmet needs, and optimizing existing treatments. As a matter of fact, diabetes care and the means of the delivery of biologics has evolved dramatically in the past few years. While injections were the only means for the successful delivery of biologics in the 1920s, in the recent decades, various novel approaches for oral delivery have emerged. These include the utilization of permeation enhancers, formulation approaches, and the use of emerging drug-device combinations. We look at such an evolution in the next section as we anticipate that a similar progress in gene delivery is to be expected in a not-too-distant future.

## 3. Evolution of Macromolecule Delivery

### 3.1. From Injections to Oral Delivery of Macromolecules

A century has gone by since the first documented experiment where a biologic was administered orally [120]. In this study, the pathologist Geoffrey Harrison dosed four patients with an alcoholic solution containing insulin, but only one of the patients showed a significant decrease in the glucose levels. These results are of no surprise when looking at the complexity of the absorption mechanisms of the gastrointestinal tract. The harsh environment of the gastrointestinal tract including its high enzymatic activity and abrupt pH triggers drug degradation and writes off the drug’s pharmacodynamics. Moreover, the impermeability of the intestinal cells to large molecules and the diffusion impairment posed by the mucin layer contribute to hindering the absorption into the bloodstream of peptides, proteins, monoclonal antibodies, and other large molecules [121]. For this reason, injections and intravenous infusions are needed to ensure that they reach systemic circulation unaltered.

For decades, scientists have explored various excipients and delivery technologies to diminish the degradation in the gastrointestinal tract and to increase permeability across the enterocytes, with the purpose of enabling the oral administration route for macromolecules. In the 80s, oral cyclosporin A and desmopressin entered the market. However, these are exceptions that are largely based on unique physicochemical characteristics of the two peptides. Last year, two more oral biologics were approved: Rybelsus^®^ from Novo Nordisk, an oral semaglutide tablet, and Mycappsa^®^ from Chiasma, an oral octreotide capsule. Although the oral bioavailabilities of semaglutide and octreotide are low—in the range of 0.5–2%, their success holds great promise for the oral delivery of biologics in the future [122,123,124]. Rybelsus^®^ and Mycappsa^®^ utilize permeation enhancers (sodium salcaprozate (SNAC) and sodium caprylate) that have been thoroughly explored over many years to facilitate oral absorption of macromolecules such as peptides and oligonucleotides. Permeation enhancers enable para- and/or transcellular transport of the macromolecules by loosening the tight junctions between the enterocytes or fluidizing the cell membrane, respectively (reviewed in [125]). In Rybelsus^®^, the permeation enhancer SNAC increases the permeation of semaglutide over the gastric barrier by a transcellular mechanism of action [124,126]. Furthermore, SNAC has been shown to neutralize the low pH of the gastric fluids, which can attenuate the enzymatic activity in the stomach. SNAC also enables the release of monomers of semaglutide from the more stable multimeric form in the tablet. To succeed at raising permeation, permeation enhancers need to be administered simultaneously with the drug or shortly before [124,127].

In Mycappsa^®^, the cyclic octa-peptide, octreotide, has been formulated as a suspension in oil by spray drying an aqueous solution of octreotide, polyvinylpyrrolidone, and sodium caprylate, and suspending the spray-dried particles in a mixture of tricaprylate and dicaprylate [127,128]. It has been shown that sodium caprylate is an essential part of the formulation in order to achieve absorption [128]. Another similar medium chain fatty acid salt, sodium caprate (C10 fatty acid) is the most widely explored permeation enhancer for the delivery of macromolecules, however, it has never proceeded past phase II for the oral delivery of peptides [129]. Several clinical studies have been performed using the permeation enhancer for peptides: desmopressin, low molecular weight heparins, and more recently, insulin 338 (long-acting basal insulin), also with low bioavailabilities of up to single digit percentages [130,131]. In addition, a number of clinical studies are performed using other permeation enhancers and stabilizers/protease inhibitors (e.g., lauroyl carnitine or taurodeoxycholate combined with citric acid (Peptelligence technology by Enteris Biopharma) or bile salts/EDTA/protease inhibitor/oils (POD^TM^ technology by Oramed Pharmaceuticals)). For a more comprehensive overview of oral delivery technologies, several reviews have recently been published [125,132]. Other promising emerging technologies are also being explored. Ionic liquids, consisting of a salt in a liquid state, are one such example. Pre-clinical success at delivering insulin orally has already been demonstrated, claiming a bioavailability above 50% relative to 2 insulin units/kg of a subcutaneous injection in rats [133]. An exciting prospect for ionic liquids would be its use for the oral delivery of oligonucleotides [134].

### 3.2. Oral Oligonucleotides

The first oral oligonucleotide to progress to the clinic is credited to Ionis Pharmaceuticals (San Diego, CA, USA, previous name ISIS). They have advanced an oral antisense molecule (ISIS 104838) into phase I using a sodium caprate-based tablet, achieving somewhat higher oral bioavailability values than peptides—an average of 9.5% plasma bioavailability across four formulations tested [135]. More recently, Ionis Pharmaceuticals published, together with AstraZeneca, the oral delivery of an oligonucleotide conjugated to GalNAc (AZD8233, also known as ION-863633) for targeting of PCSK9 in the liver, also using a sodium caprate-based tablet [112]. The exposure was measured in cynomolgus monkeys, and a bioavailability of 7% in the liver was achieved, which was 5-fold higher than measured in plasma. In December 2020, Ionis Pharmaceuticals and AstraZeneca announced that they had discontinued an ongoing phase I clinical study of AZD8233 in an oral formulation (composition unknown) because they were confident that they could improve the formulation [136]. Ionis Pharmaceuticals announced that they were expanding its oral delivery repertoire, and earlier this year, they teamed up with Progenity to use their oral biopharmaceutical delivery system—described in a later section—for the oral systemic delivery of ASOs [137].

As of July 2021, no further orally-delivered oligonucleotides have been registered at clinicaltrials.gov. However, the number of research studies with a focus on delivering oligonucleotides orally is burgeoning [138,139]. Alnylam Pharmaceuticals, in 2019, achieved a proof of concept for the oral dosing of GalNAc-siRNA conjugates. A sustained knockdown effect was observed for over 40 days in mice following three doses at 3 mg/kg administered via oral gavage [140]. More recently, using a non-viral vector platform, namely nanoparticles, enGene has delivered nucleic acid-based cargo into the epithelial cells of the intestine with the purpose of using the cells as bioreactors for therapeutic peptides or proteins [111]. By using this approach, a different pharmacokinetic local or systemic profile of the therapeutic peptide can be obtained. Their nanoparticle platform could then be encapsulated into a Gene Pill for targeted delivery to specific sections of the small intestine defined by tuning the enteric coating of the pill [111]. Oral gene therapy is also the focus of DNAlite Therapeutics, an early-stage biotechnology company leveraging a non-immunogenic proprietary vector for the oral administration of DNA and RNA cargos [141].

### 3.3. Oral Delivery of Macromolecules

Despite using permeation enhancers to enable clinically relevant exposure after oral administration of macromolecules, the exposure is low, as shown, for instance, for Rybelsus^®^ and the antisense nucleotide ISIS 104838 [124,135,142]. Low exposure may be problematic for a drug development program for various reasons: (a) it may not be possible to reach clinically relevant bioavailability of the drug, and (b) the variation in bioavailability between individuals and between dosing regimens with the same individual is usually higher for molecules with low bioavailability (for example, Rybelsus^®^ and ISIS 104838 [124,135,142,143]). Additionally, the cost of goods for the products may be high, thus, not commercially viable, as was the cause for discontinuing the development of Novo Nordisk’s oral long-acting insulin 338 [144].

The permeation enhancers that have been tested in the clinical trials have a low potency and thereby require high concentrations in each dosing unit. Rybelsus^®^ tablets contain 300 mg of SNAC per tablet, and for insulin 338 clinical trials, an amount of 550 mg of sodium caprate was used per dose [131]. Permeation enhancers have a fast absorption and elimination, as shown for SNAC and sodium caprate. The effect of the amount of water taken together with the dose as well as the timing of food intake is crucial for the exposure of the drug [123,145]. The low and variable exposure confers restrictions of the oral macromolecules that can be delivered using oral permeation enhancers. A high potency and a wide therapeutic window are needed. For Rybelsus^®^ the intraindividual variation from dose to dose in oral semaglutide bioavailability was relatively high, but as a result of daily dosing and a long plasma half-life (1 week), the variability of semaglutide exposure at steady state was reduced [123]. The aforementioned approved macromolecules, namely octreotide, desmopressin, cyclosporin A, and semaglutide range in molecular weight from 1000 g/mol to 4000 g/mol, approximately. It is noteworthy that the permeation enhancing effect decreases with increasing molecular size [127]. Accordingly, there is a major request for alternative technologies that can address these many challenges. Some of the latest research solves the challenge of low bioavailability using sophisticated devices that can inject the macromolecules directly into the gastrointestinal tract.

### 3.4. Drug Device Combinations for Oral Delivery

**Figure 2:**
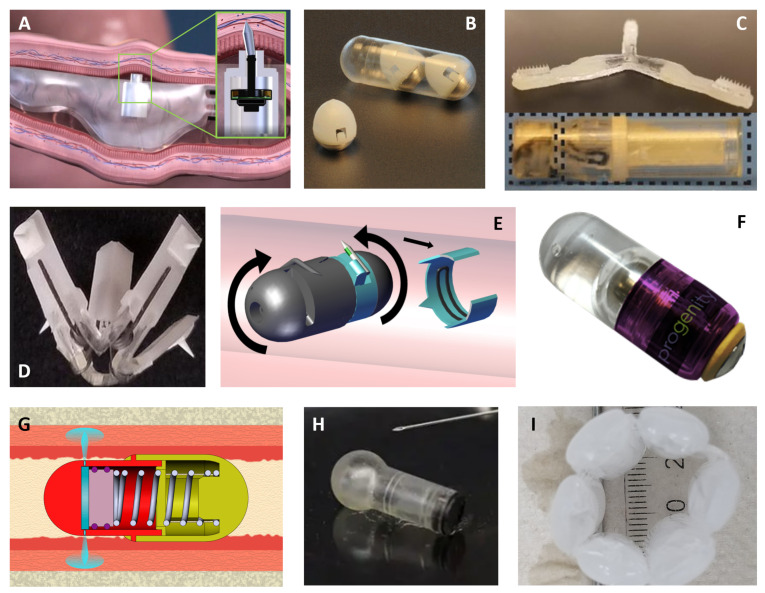
Devices designed to deliver biologics in the gastrointestinal tract. (**A**) RaniPill™ capsule delivering to the small intestine via a microneedle (Image provided by Rani Therapeutics) [147,148]; (**B**) SOMA device prototypes aimed to deliver in the stomach wall (Photo credit and copyright: Felice Frankel) [149]; (**C**) LUMI device unfolded (**top**) and inside the enteric coated capsule (**bottom**) (Adapted with permission from [150], Nature Medicine, 2019); (**D**) Flower-like device for esophageal administration [151]; E BIONDD™ device prototype for stomach delivery (Image credit Anne Lena, provided by Biograil™) [152]; (**F**) Oral biotherapeutics delivery system (Photo provided by Progenity) [153] and (**G**) JetCAP™ capsule delivers a needle-free liquid injection into the gut wall (Image provided by Baywind Bioventures) [154,155]; (**H**) MucoJet^®^ needle-free system for drug delivery in the oral cavity next to a hypodermic needle (Photo credit: Stephen McNally/UC Berkeley) [156]; and (**I**) Self-expanding device for small intestine delivery (Photo provided by Epitomee Medical) [157].

Within the last few decades, scientists have come up with various innovative solutions that have the potential to reach the holy grail of the oral delivery of macromolecules [146]. Figure 2 compiles some of these novel proprietary technologies.

Already in phase I, the RaniPill™ by Rani Therapeutics is the most advanced technology to administer biologics to the gut. Rani’s “robotic” pill consists of an enteric-coated capsule, where dissolution of the enteric-coating triggers the mixture of reactants in a balloon structure, resulting in the production of carbon dioxide (CO_2_) (Figure 2A). The CO_2_ inflates the balloon structure to deliver a drug-loaded microneedle into the wall of the small intestine where it dissolves, delivering the payload, and the rest of the pill continues its passage through the rest of the gastrointestinal tract. The device has a drug loading capacity between 1 and 3 mg. Phase I studies, where the RaniPill™ was used to administer octreotide to 52 patients, did not indicate any safety issues [147,148]. A bioavailability of 65% was reported in this study.

Scientists at the Massachusetts Institute of Technology (MIT) in collaboration with Novo Nordisk developed a turtle-inspired device named SOMA (self-orienting millimeter-scale applicator) to deliver macromolecules in the stomach wall (Figure 2B), and a luminal unfolding microneedle injector (LUMI), a star-shaped device to administer drugs to the small intestine (Figure 2C). The SOMA device rights itself within milliseconds against the stomach wall [149]. A minute after its positioning, the coating containing the spring is dissolved by the gastric fluid triggering its expansion, which, in turn, leads to the deposition of a dissolvable needle or millipost into the stomach lining. Then, the SOMA shell pursues its course to excretion. The LUMI is expelled from its enteric capsule in the small intestine following the dissolution of the enteric plunger [150]. Immediately after, the LUMI arms push the drug-loaded microneedles into the wall of the small intestine and the device remnants continue its transit through the guts undergoing partial dissolution. The LUMI, like the SOMA, demonstrated successful delivery of insulin, with a bioavailability comparable to that obtained via subcutaneous injections. In addition, the pigs dosed with these two devices did not show any blockage, tissue damage, or other signs of distress post-device administration [149,150]. A liquid version of the SOMA (L-SOMA) is also being optimized with a 4 mg drug load capacity and a drug bioavailability over 50% [158]. The same group at MIT has also investigated a temperature-responsive flower-like device that leverages elastomeric materials for its deployment (Figure 2D). Upon actuation, it delivers the drug to the esophagus wall via degradable milli needles. The ingestion of warm liquids (55 °C) triggers the retraction of the device, specifically, the shape-memory nitinol springs fold the device arms to enable its safe passage through the gastrointestinal tract, as demonstrated in preclinical experiments conducted in pigs [151]. BIONDD™ technology from Biograil™, like the SOMA, is designed to insert a drug-loaded biodegradable spike to the stomach wall (Figure 2E). BIONDD™ is manufactured by injection molding and consists of a 00 conventional capsule that attaches and delivers to the stomach wall. This technology has two parts turning around a central axis, each with a hinged spike. These two spikes are pushed out by the centrifugal force, which causes them to hook onto the stomach tissue from both sides. BIONDD™ has achieved 100% hooking efficiency in several animal studies. Moreover, this device has been validated for insulin in awake dogs, showing effective delivery to the stomach linen, equivalent to the insulin delivery obtained via subcutaneous injections. Biograil™ is currently testing its BIONDD™ platform with another four drug candidates, including oligonucleotides [152].

Progenity has developed a needle-free liquid jet, named oral biotherapeutics delivery system (OBDS), capable of delivering large molecules in the small intestine with a 400 µL liquid drug reservoir (Figure 2F). It is fabricated by injection molding the drug and biocompatible plastic components. Its actuation mechanism consists of a customizable enteric trigger. An earlier OBDS prototype loaded with human insulin, dulaglutide, and adalimumab was endoscopically placed in pigs. The bioavailability obtained for these drugs was of 19, 29, and 27%, respectively. Progenity is currently conducting preclinical studies with the fully autonomous device [153]. Baywind Bioventures Propel Biologics™ JetCAP™ proprietary technology, like the OBDS, uses an enteric-coated tablet actuator to trigger a needle-free injection to deliver drugs into and onto the small intestinal wall (Figure 2G). The system was modelled based on the fluid dynamics of a pre-filled syringe. The JetCAP™ deployment starts with the dissolution of an enteric coated tablet (actuator), which is holding a spring in the prone position. The nozzles used to create the fluid stream are also enteric coated to seal them during storage. Upon entering the upper gut, the tablet dissolves, releasing the spring, which expands to push the plunger. The plunger then pushes the liquid formulation at high velocity into the gut wall, completing the needle-free injection. The device is currently the size of a 000 capsule and can deliver up to 30 mg per dose. Preclinical pharmacokinetic experiments are being conducted in dogs [154,155].

Ultrasound and iontophoresis are two of the physical mechanisms being investigated to disrupt the striking function of biological membranes and to spur drug delivery. Notably, the former technique has been widely scrutinized to deliver insulin across the skin [159]. Recently, a drug-device system integrating ultrasound has successfully administered drugs to the oral mucosa to treat oral inflammatory lesions in hamsters [160]. Tolerability of this device without the drug cargo was confirmed in unanesthetized dogs. Biologics such as insulin and human growth hormone have also been dispensed to the oral cavity of pigs via dissolving microneedles using a custom-made applicator. Furthermore, a clinical study where drug-free microneedle patches were applied to different locations in the oral cavity of a hundred human volunteers acknowledged the preference of such patches over hypodermic injections [161]. Additionally, targeting the oral mucosa, MucoJet^®^ is a needle-free drug delivery device validated in rabbits for the oral administration of vaccines in a non-invasive manner (Figure 2H) [156]. The next generation of the MucoJet^®^ device aiming for small intestine delivery is under development.

Intestinal polymeric patch systems have been employed to deliver insulin either alone or in combination with iontophoresis [162,163]. In particular, patches have been delivered inside enteric-coated capsules, which adhered to the intestinal epithelium thanks to their mucoadhesive properties. Their insulin payload is released over 30 min in rats [162]. The impermeable layer located on one side of the patch conferred unidirectional insulin release toward the epithelium. Permeation enhancers and peptidase inhibitors increased absorption and protected insulin from the harsh environment, respectively. A 3-fold increase in the insulin delivered across intestinal cells was noted for similarly prepared patches excluding the permeation enhancer when combined with an intermittent electric current [163]. Epitomee Medical’s shape-shifting device concept leverages the mucoadhesive properties of its external layer to attach to the intestinal wall and deliver the drug cargo (Figure 2I). Liquid absorption by the super absorbent polymers triggers device self-expansion, which pushes the mucoadhesive drug-loaded layer against the small intestine, where it adheres and releases the embedded drug [157]. Other multi-layer microdevices have been fabricated via inkjet printing with a reservoir containing insulin. This technique, yet to be tested *in vivo* for the delivery of biologics, allows for an efficient and stable drug loading. The microdevice geometry facilitates unilateral insulin release toward the intestinal epithelium *in vitro* [164]. The same group designed nanostraw microdevices that adhere to the intestinal epithelial tissue, control drug loading and release, and limit the payload exposure to the environment. The nanostraw technology has been validated *ex vivo* for insulin on murine intestinal tissue [165]. Even at an earlier research stage, microcontainers are being investigated for oral insulin delivery in conjunction with permeation enhancers [166]. While this delivery method showed promising permeation results in a human epithelial cell line, no insulin absorption was noted upon oral gavage of insulin-loaded microcontainers. Other oral devices include electronics, which enable them to administer drug molecules in response to the real-time feedback provided by the device [167,168]. An example of this is IntelliCap^®^, a certified medical device consisting of a 000-capsule containing temperature and pH sensors, a liquid-drug reservoir, a microprocessor, batteries, a wireless transceiver, and a stepper motor for drug release [169]. Nonetheless, these electronic-based devices despite enabling controlled drug release in response to the pharmacokinetic data obtained via monitoring *in vivo*, are not yet suitable to deliver biologics since the drug is released in the stomach fluid and is, therefore, prone to degradation.

The last five years have seen a surge for innovative ingestible devices for drug delivery. Not only do these novel devices enable the oral administration of macromolecules such as insulin, but they also lay the foundation for the exploration of oral delivery of the next generation of gene and oligo therapies, potentially even CRISPR systems in a distant future.

## 4. Forward Looking

Treatments that years ago did not exist such as insulin are now commodities. Breakthroughs in regenerative medicine have come at a fast pace in the last few years and while gene therapies in dermatology have yet to reach the market, many are showing promising results in clinical trials. Hence, it is a matter of time before gene and oligo therapies also become prevalent. This would be a quantum leap for patients suffering from incurable skin diseases caused by gene mutations. For this to occur, drug delivery systems will have to evolve accordingly to overcome the current challenges associated with the delivery of these therapies. The first oral oligo therapies are just entering clinical trials, as described in this review. Having gene and oligo therapies delivered orally would be a significant advantage for patients. However, for this to become a reality, the strategies to protect the cargo in the gastrointestinal tract, increase the absorption over the intestinal barrier, and target specific tissues need to evolve. Notwithstanding, with the increasing number of oral devices in development and clinical trials that show success in delivering macromolecules orally, it is to be expected that the armamentarium of oral devices in the clinic will increase in the future. We, accordingly, envision that gene therapies will also be available for ingestion in orally-dosed capsules.

## Figures and Tables

**Table 1:** Ongoing and completed clinical trials using gene therapy to treat dermatological conditions [47,48].

Skin Disease	Clinical Trial	Phase	Company	Biological	Administration Route	Treatment	Estimated Completion Date
***Ex vivo*** **approaches**							
**Netherton’s syndrome**	NCT01545323	I	Great Ormond Street Hospital for Children NHS Foundation Trust	N/A	Skin graft	Autologous epidermal sheet graft from *ex vivo SPINK5* gene-corrected keratinocyte stem cells using a lentiviral vector	April 2018
**JEB**	NCT03490331/2016-000095-17	I/II	Holostem Terapie Avanzate	N/A	Skin graft	Autologous cultured epidermal grafts genetically corrected with gamma-retroviral vectors carrying *COL17A1*	December 2021
**RDEB**	NCT02984085	I/II	Holostem Terapie Avanzate	N/A	Skin graft	Autologous cultured epidermal grafts genetically corrected with gamma-retroviral vectors carrying *COL7A1*	December 2020
**RDEB**	NCT02493816/2014-004884-19	I	King’s College London	N/A	Skin graft	Intradermal injection of SIN lentiviral virus-mediated *COL7A1* gene-modified autologous fibroblasts in adults	March 2018
**RDEB**	2016-002790-35	I/II	INSERM	N/A	Skin graft	Autologous skin equivalent grafts genetically corrected with a *COL7A1*-encoding SIN retroviral vector	Unknown
**RDEB**	NCT04186650	I/II	Institut National de la Santé et de la Recherche Médicale	N/A	Skin graft	Autologous skin equivalent grafts genetically corrected with a *COL7A1*-encoding SIN retroviral vector	September 2021
**RDEB**	NCT01263379	I/II	Stanford University (with NIAMS and Abeona Therapeutics)	LZRSE	Skin graft	*COL7A1* engineered autologous epidermal sheets transfected *ex vivo* using a retrovirus	December 2025
**RDEB**	NCT02810951	I/II	Castle Creek Pharmaceutical	FCX-007	Intradermal injection	Genetically modified autologous fibroblasts to produce type VII collagen	December 2033
**RDEB**	NCT04213261	III	Castle Creek Pharmaceutical	FCX-007	Intradermal injection	Genetically modified autologous fibroblasts to produce type VII collagen	December 2036
**RDEB**	NCT04227106	III	Abeona Therapeutics	EB-101	Skin graft	Autologous RDEB keratinocytes isolated from skin biopsies and transduced with a recombinant retrovirus containing *COL7A1*	April 2022
***In vivo*** **approaches**							
**ARCI**	NCT04047732	I/II	Krystal Biotech	KB105	Topical	Replication-defective, non-integrating HSV-1 expressing human transglutaminase 1	March 2025
**DEB**	NCT03536143	II	Krystal Biotech	B-VEC (previously KB103)	Topical	Replication-defective, non-integrating HSV-1 expressing human type VIII collagen	March 2024
**DEB**	NCT04491604	III	Krystal Biotech	B-VEC (previously KB103)	Topical	Replication-defective, non-integrating HSV-1 expressing human type VII collagen	August 2021
**Hypertrophic scar**	NCT04540900	I	Krystal Biotech	KB301	Intradermal injection	Replication-defective, non-integrating HSV-1 expressing human type III collagen	January 2022

ARCI: autosomal recessive congenital ichthyoses, NIAMS: National Institute of Arthritis and Musculoskeletal and Skin Diseases, DEB: dystrophic epidermolysis bullosa, RDEB: recessive DEB, JEB: junctional epidermolysis bullosa, HSV: herpes simplex virus, SIN: self-inactivating, N/A: not applicable.

**Table 2:** Ongoing and completed clinical trials using oligonucleotides to treat dermatological conditions [47,48].

Skin Disease	Clinical Trial	Phase	Company	Biological	Administration Route	Treatment	Estimated Completion Date
AD	NCT02079688	II	Sterna Biologicals GmbH & Co. KG	SB011	Topical	DNAzyme hgd40 targeting GATA3, a highly mutated transcription factor	January 2017
DEB	NCT03605069	I/II	Wings Therapeutics	QR-313	Topical	21-nucleotide ASO designed to hybridize to a specific sequence in the *COL7A1* pre-messenger RNA	September 2020
Hypertrophic scar	NCT02956317	I/II	Sirnaomics	STP705	Intradermal injection	Two siRNA oligonucleotides, targeting TGF-β1 and Cox-2 mRNA, respectively, formulated in nanoparticles	January 2018
Hypertrophic scar	NCT02205476	II	Pfizer	PF-06473871	Intradermal injection	Anti-CTGF antisense oligonucleotide	January 2015
Hypertrophic scar	NCT02030275/NCT02246465	II	RXi Pharmaceuticals	RXI-109	Intradermal injection	Self-delivering RNAi compound targeting CTGF	June 2016
Hypertrophic scar	NCT04012099	II	Hugel	BMT101	Intradermal injection	Cell penetrating asymmetric siRNA targeting human CTGF	August 2022
Hypertrophic scar	NCT04877756	II	Olix Pharmaceuticals	OLX10010	Intradermal injection	Cell penetrating asymmetric siRNA targeting human CTGF	March 2023
PC	NCT00716014	I	Pachyonychia Congenita Project	TD101	Intralesional injection	siRNA designed to target a mutation of the PC keratin K6a	August 2008
Psoriasis	Unknown	I	Purdue Pharma, Exicure	AST-005	Topical	Nanoparticle-based SNA to knockdown a tumor necrosis factor gene	August 2016

AD: atopic dermatitis, ASO: antisense oligonucleotide, CTGF: connective tissue growth factor, DEB: dystrophic epidermolysis bullosa, PC: pachyonychia congenita, SNA: spherical nucleic acid.

## Data Availability

No new data were created or analyzed in this study. Data sharing is not applicable to this article.
